# Value of Vaccinations: A Fundamental Public Health Priority to Be Fully Evaluated

**DOI:** 10.3390/vaccines13050479

**Published:** 2025-04-29

**Authors:** Sara Boccalini

**Affiliations:** Department of Health Science, University of Florence, 50134 Florence, Italy; sara.boccalini@unifi.it

**Keywords:** efficacy, cost, cost-effectiveness, investment, savings, return of investment, antimicrobial resistance, fiscal impact, health technology assessment, intangible benefits

## Abstract

Introduction: Vaccinations are one of the most impactful public health interventions, saving millions of lives annually and reducing the spread of infectious diseases. Numerous vaccines are expected to become available in the future. Decision-makers will have to thoroughly evaluate them. It is essential to fully comprehend the value of vaccinations to effectively and efficiently guide decisions. Methods: This work aims to highlight the multifaceted benefits of vaccination, extending beyond clinical outcomes to encompass profound economic and societal advantages. Results: Vaccinations should be considered an investment, not a cost. In comparison to other health expenditures, the vaccine costs can be considered moderate. Vaccinations can also reduce the fiscal burden by avoiding diseases, minimizing lost workdays and absenteeism, lowering disability claims, and increasing workforce productivity. The costs of non-vaccination represent a relevant issue. Vaccination also plays a key role in addressing the global challenge of antimicrobial resistance. Apart from quantifiable economic parameters, vaccines also have intangible benefits reducing pain and avoiding quality of life lost and deaths. Conclusions: Comprehensive Health Technology Assessments are required to understand the overall value of vaccinations.

## 1. Introduction

Vaccinations are undoubtedly one of the most impactful public health interventions in history. In 1798, Jenner used animal poxvirus to prevent smallpox based on the idea that an agent virulent for animals might be attenuated in humans. Since then, many types of vaccines (e.g., attenuated, inactivated, reassortant) have been developed in laboratories, and in the 20th century, they were developed based on immunologic markers [1,2,3]. Vaccines have led to significant reductions in infections and diseases, wherever they are applied. As a matter of fact, much evidence highlights that vaccinations are a crucial public health measure, offering significant clinical benefits. They save millions of lives annually, mitigate and reduce the spread of infectious diseases, and improve the resilience of healthcare systems worldwide. However, the true and comprehensive value of vaccines is often misunderstood or underestimated by the general population, key stakeholders, and decision-makers.

As a matter of fact, the clinical benefits of vaccination—such as the prevention of disease cases, complications, hospitalizations, and deaths—are not adequately recognized. Unlike drugs, vaccines are given to healthy people with the aim of not making them ill. In this case, the benefit of vaccination (i.e., the number of cases of disease avoided by immunization) is not perceived by the population. This issue can hinder vaccination uptake. On the contrary, vaccines are often perceived only as a relevant cost (the cost of purchase and administration of doses) impacting limited public health resources. However, numerous scientific studies contradict these concerns, highlighting the cost-effectiveness and long-term economic advantages of vaccination.

Numerous vaccines have been developed over the past decades, and many more are expected to become available in the future [4,5,6,7,8,9]. As new vaccines demonstrating safety and efficacy emerge, decision-makers will have to thoroughly evaluate them before making recommendations for their inclusion in national or regional immunization programs. It is therefore essential to fully comprehend the value of vaccination to effectively and efficiently guide decisions regarding the introduction and management of new vaccination strategies.

**Figure 1:**
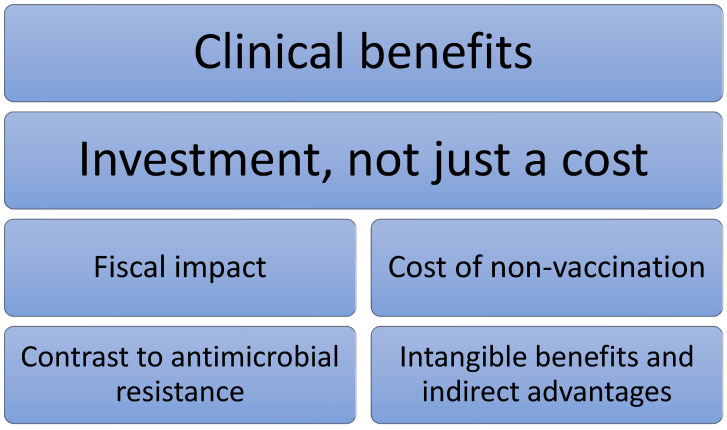
The multifaceted benefits of vaccination.

This work aims to highlight the multifaceted benefits of vaccination, extending beyond clinical outcomes to encompass profound economic and societal advantages (Figure 1).

## 2. Clinical Benefits: The Foundation of Vaccine Advocacy

The clinical efficacy and effectiveness of vaccines are beyond question, with a wealth of scientific evidence demonstrating their ability to reduce disease incidence and prevalence, prevent severe health complications, and save lives. Concerning the clinical benefits of vaccination, vaccines are considered the health intervention with the greatest impact on reducing mortality and controlling population growth, second only to access to safe water [10,11,12,13,14,15,16,17,18].

The undeniable clinical benefits of vaccines are particularly relevant for vulnerable populations, including older pregnant women, individuals at risk for co-morbidity, those with occupational activities, or those with certain behaviors or living conditions (drug users, men who have sex with men, etc.). For some of these groups, vaccination often serves as a lifeline.

Despite these clear clinical benefits, the public’s perception of vaccines remains a persistent challenge. Many individuals, even in well-informed societies, underestimate the importance of immunization, focusing instead on isolated risks of adverse reactions or misinformation concerning vaccination. This gap in understanding not only hinders the achievement of vaccination coverage targets but also undermines the collective effort required to achieve herd immunity and protect the most vulnerable subjects.

## 3. Vaccinations Are an Investment

One of the most pervasive misconceptions about vaccination is that it represents only a financial burden rather than a strategic investment in health. As a matter of fact, vaccinations are frequently viewed as a cost, considering the expenses associated with dose procurement, administration, and organizational logistics. This perception is amplified by the increasing cost of advanced, highly technological next-generation vaccines. On the contrary, the economic analysis of vaccination strategies tells a different story: vaccinations are an investment, not a cost. In addition, this investment could be considered long-term if the vaccinees are children who have a high life expectancy.

The costs of vaccine programs can appear high but are cheaper than those of other drugs. For example, in Italy, the recommended vaccines included in the National Immunization Plan (NIP) are supplied free of charge to the target population. In this country, public expenditure on vaccines more than doubled between 2014 and 2023, increasing from EUR 4.8 to EUR 12.1 per capita. However, the cost per capita could be considered irrelevant when comparing the possible disease burden that can be avoided after immunization. At the same time, if the total public expenditure on vaccines appears particularly relevant (EUR 712.2 million), it accounts for only 2.7% of the overall public health budget (EUR 24,881 million) [19]. Therefore, in comparison to other health expenditures, the vaccine costs can be considered moderate.

The cost of a vaccination program can be assessed using various approaches with different degrees of complexity and detail. Fernandez Conde et al. compared healthcare costs and vaccination costs in the Spanish setting. According to the 2023 NIP, vaccinating a healthy individual throughout their lifetime, from birth to the age of 83 years, costs approximately EUR 1500 (EUR 1542 for women and EUR 1498 for men). For individuals with chronic illnesses, the cost rises to between EUR 1735 and EUR 3160. With total healthcare spending amounting to EUR 115.5 billion, the estimated annual cost of vaccinations covered by the 2023 NIP, assuming complete coverage, is around EUR 565 million. This amounts to roughly 23% of the funding that goes to preventive care and public health services but represents only 0.5% of total healthcare spending. Immunization is therefore a minimal investment considering the large decrease in disease burden that it achieves. For instance, the average treatment costs for influenza and meningitis cases amount to EUR 3276 and EUR 9712, respectively [20].

Moreover, unlike most other health interventions, nearly all economic evaluations of new vaccine introduction or vaccine strategies demonstrated a favorable cost-effectiveness profile, particularly if the vaccination targets at-risk groups [21,22,23,24,25,26,27].

In most instances, immunizations are also cost-saving and have a return on investment (ROI) that is several times the initial costs. For example, in Italy, NIP for children has demonstrated the extensive benefits of continued investment in childhood vaccinations, such as decreased disease-related morbidity, mortality, and costs. It is estimated that the pediatric NIP has averted 1.8 million cases of vaccine-preventable disease (VPD) and saved 3330 lives, which corresponds to 45,900 fewer years of life lost and 57,000 fewer QALYs lost. The EUR 285 million spent on vaccination is more than offset by the EUR 1.6 billion in disease-related cost savings, yielding a return on investment of 1.7 from the healthcare sector’s perspective and 5.6 from a societal perspective. When the value of QALYs gained is included, the return on investment rises to 15.6 [28].

In this context, there are many other examples. In 1991, Italy was one of the first countries worldwide to introduce a universal hepatitis B vaccination for children. Since then, epidemiological data have clearly demonstrated the huge clinical benefits of the vaccination, with a great reduction in the incidence of HBV cases. According to an economic a posteriori evaluation, during the first 30 years, the implementation of universal HBV vaccination in Italy resulted in a cost-saving strategy, and more advantageous effects will be further achieved after these first decades. The study estimates a big drop in HBV-related diseases (−82% in infections, chronic disease, and hepatocellular carcinoma cases), and related costs (−67% in the immunization period and −85% in 2021–2070) attributable to vaccination. The return on investment is >1 (1.3) for the first thirty-year immunization period and is predicted to almost triple the economic savings in the period 2021–2070, both for the National Health Service (NHS) (2.74) and from societal perspectives (2.75), considering all chronic HBV cases avoided by vaccination in a long-time horizon. The break-even point was already achieved some years ago for the NHS and for society, and since then, more and more money has been progressively saved. This favorable economic profile continues to be current even though vaccination has greatly reduced the incidence of HBV infections [29].

At an international level, a recent study pointed out that vaccination programs for adults can generate socio-economic returns of up to 19 times their initial cost when accounting for the complete set of benefits to individuals, healthcare systems, and society in general. Specifically, the study, conducted by the Office of Health Economics (OHE), reviewed four vaccination programs—against influenza, pneumococcal infection, respiratory syncytial virus (RSV), and herpes zoster—in ten nations with different healthcare systems. The cost analysis determined that vaccinations in adults could yield net societal benefits amounting to billions of dollars or approximately USD 4637 for the full course of vaccination in each subject. The report also indicates that adult immunization can provide socio-economic returns comparable to those arising from vaccination programs among children [30].

Although vaccines may be a good health investment overall, it cannot be presumed that every nation and every person will have the means and resources to achieve this goal. In certain countries, the expense of vaccination can be extraordinarily prohibitive. In these countries, individuals will remain unvaccinated unless their government or other global health entity can provide assistance to low-cost or free immunizations. Therefore, it is essential to address vaccination issues globally.

## 4. Discussion

If vaccinations are an investment in health and money, they also have other important values.

### 4.1. Fiscal Impact: Ripple Effect

Vaccinations are a long-term investment in public health and can be economically sustainable. In addition, vaccinations can also reduce the fiscal burden by avoiding diseases, minimizing lost workdays and absenteeism, lowering disability claims, and increasing workforce productivity. These factors result in fiscal gains that frequently greatly exceed the original expenditure by a significant margin. For example, vaccination could have fiscal benefits of up to twice the investment per capita and as much as 16 times in terms of avoided productivity losses, as reported by Ruggeri et al. for three vaccination strategies against influenza, pneumococcal disease, and herpes zoster in Italy [31]. This ripple effect strengthens not only individual livelihoods but also national economies. A healthy population is one of the primary drivers for a nation’s economic and social development, which boosts productivity, with a larger workforce, higher wages, increased consumption, and savings [32,33,34,35,36]. Conversely, the COVID-19 pandemic has demonstrated how an economic crisis can result from a health crisis [37]. Therefore, vaccines significantly contribute to the well-being of society by reducing disease transmission, improving quality of life, and building community resilience.

### 4.2. Cost of Non-Vaccination

On the other hand, the costs of non-vaccination are certainly not taken into consideration in the decision-making process, but they represent a relevant issue [38]. For example, between 2017 and 2021, approximately 1.2 million adolescents in Italy were not adequately vaccinated against HPV, leading to a projected clinical cost of over EUR 905 million. Achieving a 95% coverage rate could reduce costs by EUR 529 million, even after accounting for vaccine expenses. Therefore, the lack of HPV immunization during the COVID-19 pandemic period in Italy was an opportunity loss with subsequent incremental costs for the Italian NHS [39]. This economic impact of non-vaccination is more relevant if non-vaccinees are part of vulnerable populations. These groups are often the hardest to reach but face the highest disease burden of preventable diseases.

### 4.3. Role of Vaccines in Combating Antimicrobial Resistance (AMR)

Vaccination also plays a key role in addressing the global challenge of antimicrobial resistance. Vaccines reduce the need for antibiotics and consequently decrease the emergence of resistant strains, directly or indirectly preventing bacterial and viral infections. According to a recent technical report by the WHO on the impact of vaccines in reducing antimicrobial resistance and antibiotic use, vaccinating against 24 pathogens could reduce global antibiotic usage by 22%, or 2.5 billion daily doses annually. This reduction has the potential to lower hospital treatment costs for resistant pathogens, currently estimated at USD 730 billion per year, by one-third. Such benefits underscore the dual role of vaccines in both disease prevention and safeguarding the efficacy and efficiency of existing treatments [40].

### 4.4. Other Intangible and Misunderstood Values of Vaccines

Apart from quantifiable economic parameters, vaccines also have intangible benefits: pain, quality of life lost, and deaths avoided due to vaccination cannot be quantified in terms of money, but they have a huge value [41].

In addition, the indirect impact of immunization (herd immunity) on society is an often-neglected or incompletely measured benefit of vaccination. The decrease in disease incidence and prevalence due to vaccinations enables the redistribution of healthcare resources, which can be invested in other priorities in healthcare, thereby increasing the sustainability of health systems. In particular, the economic savings arising from vaccination programs can be utilized to pay for innovative treatments, invest in infrastructure, and finance other public health priorities, thereby making healthcare systems sustainable for generations to come.

### 4.5. Health Technology Assessment (HTA): A Tool to Reveal the Overall Vaccine Value

Comprehensive Health Technology Assessments are required to understand the overall value of vaccinations: HTA is the key approach for illustrating the comprehensive value of vaccinations. HTA involves the systematic collection, analysis, and synthesis of scientific evidence on different aspects of technology (the health problem and current use of technology; the characteristics of technology; safety; efficacy/effectiveness; cost and economic valuation; and organizational, ethical, social, and, finally, legal aspects) [42]. HTAs enable policymakers to prioritize immunization programs, make well-informed decisions, and effectively manage resources by giving a comprehensive perspective to payers and society. The contribution of HTA to the evaluation of vaccines is not in doubt, as attested by the WHO policy on new vaccine introduction [43]. Moreover, European Regulation (EU) 2021/2282 on HTA [44] emphasizes HTA as a tool for enabling the timely introduction and use of new technologies, with a view to enhancing sustainability and fostering innovation. Preventive interventions, including vaccines, are among the health technologies covered in the regulation. Nevertheless, even if scientific evidence is collected in HTA reports, it may not be sufficient for applying HTA in the decision process. For example, HTA was applied many times with different purposes in the evaluation of vaccinations in Italy: assessment of new vaccines and vaccination strategies or their re-assessment following new evidence or indications of use. However, it was not always coincided with the timing of the vaccine marketing authorization and the release of national recommendations [45].

## 5. Conclusions: Recognizing Vaccines as a Public Health Priority

Vaccines offer much more than the prevention of illness. They are also an essential public health investment because they yield massive clinical, economic, and social advantages. They contribute to healthcare efficiency, economic sustainability, and combating international health security threats like AMR. Through reducing healthcare costs, delivering equitable health outcomes, creating budgetary sustainability, and combating AMR, their impact cannot be matched. As we look ahead to the future, it is imperative that we fully recognize the opportunities that vaccinations offer. This recognition will not only improve healthcare systems but also lead to a healthier and stronger population for generations to come.

However, vaccines can be effective and efficient if they are administered properly to the target population. Therefore, a key point is encouraging the acceptability of vaccinations through specific promotion and training activities and making vaccines accessible to various populations worldwide. In addition, in future years, the control over and struggle against vaccine hesitancy, vaccine fatigue, and resistance to the administration of numerous vaccines (over-vaccination) in many populations and locations should be included in vaccination strategies.
